# Grundriss der Pathologischen Semeiotik, zum Gebrauche bei Vorlesungen

**Published:** 1838-10-01

**Authors:** 


### Grundriss der Pathologischen Semiotik, zum Gebrauche bei Vorlesungen. Von *A. F. Schill*, M.D. Tubingen, 1837.

### Outlines of Pathological Semeiology, &c.

"Si non vis intelligi, debes negligi," is a maxim by the spirit and letter of which the Editors of this Journal invariably endeavour to regulate their selection of those works which are to constitute the subject of analysis in its pages. To the observance of this rule they have been determined by several motives, among which a respect for the judgment of their readers, and a desire to spare their time, and to abstain from putting their temper and patience to too severe a test, are not the least influential. To the Editors it makes not a whit of difference under what garb or in what language trash and absurdity may be conveyed—these ingredients, which truth obliges us to say, so frequently constitute the bulk of many a ponderous medical work imported into this country from our continental friends, cannot be changed by any alchemistical influence of the language in which they may be presented. In spite of the unwieldy stiffness of barbarous Latin, the tawdry tinsel of French frippery, or the pompous pedantry of German sciolism, nonsense will be nonsense still; and whilst they envy not the taste of those journalists, who seem determined to stimulate the stomachs of their readers by the high-seasoned fricassees of continental cookery, they shall continue, as has been their wont, to give the preference, when preference is merited, to the wholesome and substantial pabulum to be obtained from the intellectual stores of old England. The object of the writer of these remarks is to answer a charge, which some have advanced against this Journal, viz. that it is too sparing in the foreign review department. That it is not and that it has not been negligent in the department of reviewing foreign works, where these possessed merit, we could adduce many, very many proofs. To mention one out of several, (and certainly the Medico-Chirurgical may take no small praise to itself for the fact), it was to the extended and frequent notices and strong recommendations contained in it of Laennec's work on auscultation, that the so early introduction into this country of that splendid auxiliary of modern pathology may in a great measure be attributed. We could cite other instances of a similar kind; but we trust it is unnecessary; we again repeat it, that whenever we shall find sufficient merit in foreign medical literature, we are determined to give our readers the earliest and fullest notice possible of it; once more however we say we will not countenance nonsense merely because it assumes a foreign dress.

In conformity with this our rule, we now present to our readers a short analysis of a work which has lately appeared in Germany on semeiology; we are the more disposed to do so, as the author not only appears to be one of the very few of his countrymen who write with a desire of being understood, but also because we conceive his book to be a very good one. Of this, however, our readers will be able to form a judgment for themselves from the extacts we shall present. Strange to say, we have not in our language any work exclusively devoted to the subject of semeiology. This branch of medicine among the ancients, and indeed until within the last 20 years, was but very imperfectly cultivated; its only bases formerly were physiology and clinical observation; and though such foundations may have sufficed for nearly every useful purpose as connected with *prognosis*, they were altogether insufficient for the formation of anything like a certain *diagnosis*, which generally speaking can only be established by comparing the phenomena observed during life with the lesions found on the dead body. The falsity of the assertion that the disturbance of a function, indicates lesion of the organ which presides over that function is proved daily by the researches of pathological anatomy. So that it is to clinical observation, physiology, and pathological anatomy, all three combined, that it belongs to determine the real value of the signs of diseases.

The author commences his work by an introductory chapter, in which he lays down, §. 1, the signification he attaches to the term semeiology; §. 2, the sources of this department; §. 3, the mode of forming signs; then the relation of semeiology to the other pathological sciences, §. 8-10. Value of semeiology, and its history, §. 12-20.

Semeiology he defines to be "the doctrine of the relations in which the phenomena in the human system stand with respect to the vital state which produces them." According as the latter is normal or morbid, semeiology is divided into physiological and pathological. Pathological semeiology considers the phenomena, whether normal, or abnormal, according to their relations to the states of disease, which have produced them; it includes within the circle of its enquiries those changes which the functions present, as well as all changes of the organs, as far as they stand open to observation in the living body. With respect to the *sources* of semeiology, observation is set down as the chief, which includes clinical observation during life, and a careful notice of structural changes after death. In order that perfection may be attained in observation, it is necessary that all the organs and functions should be interrogated, as far as possible, so that whatever is found abnormal should be strictly investigated, the preceding and accompanying external influences should be carefully taken into account, and the earlier physiological and pathological phenomena be thoroughly known. The phenomena being thus carefully observed, semeiology next seeks to ascertain the cause of the individual phenomena, by enquiring with what various groups of the same phenomena they happen to be combined. It first argues from these to the changes produced in internal organs, and then from the anatomico-pathological and the pathological phenomena combined, it infers the cause which gives rise to both. The author next observes, that the diagnostic import of the individual phenomena, that is, their value in determining the present state of disease, proceeds from the more or less close connexion in which they stand with the particular species of disease as also with its seat. Here the rule holds good that, if phenomena which are connected with a disease constantly or almost constantly, stand in close relation to it, they may be accounted for or not on physiological and pathological laws. In the latter case one needs to be particularly cautious in the formation of signs, and repeated and close observation becomes requisite. The greater or less diagnostic value may be determined by enquiring on the one hand with what multifarious states of an organ a phenomenon is connected, and how great the number of affections of other organs is, which in the organ more immediately presenting the phenomenon produce the morbid state necessary thereto. On the other hand it will appear from this how different the other phenomena of this state are from each other. With respect to the prognostic value of a phenomenon, that is to be determined by the importance of the organ whose morbid state it indicates, the extent and species of the organic change, in case such be present, the degree of functional interruption occasioned by it, and lastly by the nature of the morbid process.

Semeiology, by using its materials in this way and by giving to the phenomena a diagnostic and prognostic signification, forms signs from them. Our author defines phenomenon "every change in the organism tending to disease or even within the boundaries of health, perceptible by the senses of the person observing, or the feeling of the individual observed," whilst he refers the idea of symptom to that which is perceptible in the diseased organs and functions. From the phenomena and symptoms arise signs, when they are related to a present, past, or future morbid state, so that they are connected according to physiological or pathological laws, or even according to mere experience, with some determinate species, course and seat of a morbid process. The discovery of the phenomena is the business of the senses, the formation of signs is the business of the judgment supported by experience, and requires an acquaintance with nosology. This operation has been, not without reason, considered the most difficult, and at the same time the most important in the practice of medicine. We must here observe we do not consider the author has been sufficiently explicit either on the distinction to be made between symptoms and signs, or on the analysis of those symptoms and the intellectual process of converting them into signs. And first with respect to the difference between symptoms and signs, which in medical writings have been too often confounded, we may observe, the symptom presents itself to the senses of the physician, or of the patient, that is, it is perceptible by the external senses, whilst it is only medical skill, that can translate this symptom into a sign. The perceptions of the senses would be insufficient and almost useless for the study of disease, if the intellectual faculties remained inactive, when the symptoms presented themselves to the senses. Whilst the mere application of sense is sufficient to make us acquainted with the symptoms of disease, the knowledge of the signs of disease is the result of thought and reason directed towards these same symptoms. In a word, the symptoms are in the reach of every one; but the experienced physician alone discovers on examining them the nature and value of the signs.

Galen relates an anecdote which clearly shews the distinction between sign and symptom: he relates that he was at one time dangerously ill, and having overheard two friends of his, who were standing near his bed, discourse of certain symptoms which they had just at that moment observed on him, such as redness of face, haggard, injected, and inflamed appearance of the eyes, &c. he cried out to them instantly to adopt the necessary precautions, as he was threatened with delirium—here Galen's two friends saw the symptoms well enough; but it was only Galen himself who was able to deduce the sign of delirium from them.

Thus we see the knowledge of the symptoms is that which we acquire first in the examination of any case of disease; this knowledge we acquire by the senses. Thus by connecting and comparing these symptoms, and above all, by subjecting them, by the rules of analogy, to our previously-acquired notions of the general course of diseases, of their character, their termination, &c., we deduce from all these combined data consequences and conclusions, which are precisely what are usually and correctly called *signs*.

The conclusions, or signs, may be referred, 1st, to the knowledge of the disease itself, that is, of its nature; these are the diagnostic signs: 2nd, to the knowledge of the probable results; *i. e.* the prognostic signs. But it is only by the process of analysis we can arrive at these results. In the first place, from the number of the symptoms collected by us, we must separate those which are merely accidental from those which are essential. We must also leave out of sight those general or common symptoms which occur in almost all diseases, and which therefore do not characterize any one in particular. For this first operation it is evidently necessary to possess a profound knowledge of the character of diseases, as also of their general history; thus the study of nosology and general pathology must precede any attempt at clinical practice. When by this process of exclusion there remain in the table of symptoms those only which belong to the disease, the next thing to be done is to combine those which may lead to the discovery of the true seat of the lesion; after having done this, we have already made a considerable advance in the diagnosis, as we have now determined the generic name of the disease. We must next seek in the symptoms the essential cause of the disease; that which constitutes its nature and determines the treatment. Our next business is to seek in the combination of the symptoms whether the disease be simple or complex; if complex, to determine the nature of the complication, to judge of its importance, and to ascertain how far the treatment should be influenced by such complication.

Lastly, by combining and comparing the symptoms, by appreciating their absolute and relative severity, by considering their nature, duration, &c., we shall be able to announce the probable issue of the disease. Thus we see the chief, the most important, as well as the most difficult task of the physician, consists in discovering with promptness and certainty the nature and value of the signs obtained by the analytical process now described.

*History of Semeiology.*—Having thus far considered the import of the term semeiology, its sources, method of forming signs from symptoms, and the importance of this branch of medical science, the author next proceeds to sketch its history; in this he establishes three periods; the first dating from the time of the Asclepiades; the second extending down to the general revival of the sciences, when the study of the ancients and of observation again came into vogue; whilst the last commences from the time when pathological anatomy began to obtain influence over pathology in general, and also over semeiology. The custom prevalent among the Greeks of suspending in the temples votive tablets, containing a short description of every particular disease which presented itself for treatment, afforded to Hippocrates the materials for the first work on semeiology; this was the origin of the *Prænotiones Coacæ*, as also of the prognostics and aphorisms of Hippocrates. In the second period of its history, semeiology made but little progress. The sect, called Empirics, from whom one might expect, considering their principles, that semeiology would receive great improvements, did little or nothing for it. Galen, besides recommending the writings of Hippocrates, enriched the science of semeiology by his observations on critical days. Little more was done after this for the science till the commencement of the 15th century—with the exception of some little improvements made by the Arabians, chiefly by their contributions to the signs of individual diseases; ignorance and superstition prevailed during the latter half of this second period, urinoscopy occupying the place of semeiology. The third period produced some excellent writers on the subject of semeiology, who all, it should be observed, were ardent admirers and cultivators of the writings of the ancients. Suffice it to mention Sydenham, Baglivi, and Fr. Hoffmann, who in the 16th and 17th centuries established honorable memorials in the annals of semeiology. The 18th century is distinguished for the great progress of semeiology, which now received considerable improvements by means of some excellent commentaries on the writings of Hippocrates. The principal personages of this period were Van Swieten, De Haen, Stoll, Frank, Grant, Huxham, Jackson, Senac, Tissot, Zimmerman, cum multis aliis. The commencement of the present century has produced some good works on semeiology, as those of Landré Beauvais, Sprengel, Behrend, and Double. The last period in the history of semeiology commences with the share which pathological anatomy took in ascertaining the causes of the several morbid phenomena. By the discoveries of pathological anatomy it now became possible to detect the seat of many diseases. As more accurate experience was now attained regarding the complications of disease, men were able to separate the phenomena in the individual cases according to their various causes, and for every morbid state to find the symptoms peculiar to it. One class of diseases was enriched at this period, namely, those of the heart and lungs, by the splendid discoveries of Avenbrugger and Laennec. To the writings of these last-mentioned individuals, as well as to those of Bayle, Andral, Lallemand, Ollivier, Louis, Bouillaud, Stokes, Bright, and several others, the science of semeiology stands indebted for the proud station it now holds among the other branches of medical science. Our author next considers the subject of *signs in general*. The several morbid phenomena obtain different semeiological importance, according to the organs and functions by which they are presented, as also according to their strength and duration, and the relation in which they stand to the other phenomena. The signs afforded by the organs themselves (the pathologico-anatomical) form the smallest number. From the external superficial organs we obtain them through the sight and touch, and from those more deeply seated, through touch, percussion, and auscultation. These have great diagnostic value, as the changes in the form, size, and consistence of the organs always point out the seat of a disease. Signs derived from the functions form the chief subject of pathological semeiology; their importance varies with that of the organs whose disturbance they indicate, and its relation to the other organs.

The signs derived from the central organs are far more important than those obtained from the subordinate—the central organs sympathizing as well with those depending on them, as also with one another. Even among the central organs there is a diversity in this respect. The stomach, small intestines, and portal system, sympathize with more organs than the heart and lungs, brain and spinal cord. The stomach and small intestines will accordingly disturb the functions of other organs most in their diseases, and at the same time participate most in the diseases of other organs.

From this relation of the organs to one another, several important principles are afforded for judging of the phenomena observed in their functions. Should the morbid phenomena manifest themselves first in a central organ, and at a subsequent period, in all the other organs, or in most of those depending on it, the disease of the central organ may be inferred, more especially if the sympathetic phenomena rise and fall with those of the central organ. If the morbid phenomena shew themselves in all the organs depending on a central organ, without the latter however evincing any disturbance of function, we may infer that the seat of the disease is in the central organ in question. If they be violent in the organs connected through a central organ, the prognosis is unfavorable.

If the morbid phenomena occur chiefly in the central organ, and less intensely in the subordinate organs, the seat of disease is in the former, and the prognosis is more favorable. If, together with the central organ, only one of those subordinate to it present violent morbid phenomena, the seat of disease is to be sought in the latter, more especially if, with the increase in the violence of the phenomena in it, those of the central organ diminish. If only one central organ is affected, should the phenomena in it be of a severe character, one may infer disease of an organ sympathetically connected with it; if, on the contrary, the disturbanee is not very violent, it may indicate disease of the central organ.

The increase of the sympathetic phenomena accompanied by decrease of those proceeding from the central organ, is a sign that the disease has in this way withdrawn to the organs subordinate to it, and if these are secreting organs, that they will prove critical; if they are not, a local chronic disease may remain behind. If violent phenomena again occur in the central organs, the prognosis is unfavorable. The more subordinate an organ is, and the nearer it lies to the surface, the more favorable is the prognosis which its morbid phenomena yield. Organs of similar or physiologically-connected functions sympathize in diseases, as also those which are connected by great vessels or nerves. Accordingly in diseases of the one we shall always find phenomena present themselves in the others. In order to determine which is the diseased organ, we have to consider where the morbid phenomena are most intense, and most persevering, and retain their form most constantly, where they first occur, and on what side the dependent organs participate in the disease, or do not. Such is the mode in which we are to proceed, if organs of the same tissue present morbid phenomena simultaneously. In this case also we shall more frequently find both diseased, than only one diseased and the other sympathizing. When the morbid phenomena are violent, they may lead us to conclude that the course of the disease will be rapid. If they are very intense immediately at the onset of the disease, danger is indicated; if they increase gradually till the time of the crisis, the prognosis is rather favorable. If they have become moderate after the crisis, and then increase de novo, a return of the disease is to be dreaded. If the phenomena decrease in severity after the crisis, the transition to cure is denoted, or if they continue a long time in a slight degree, the disease has passed to the chronic form. Slight, indistinctly expressed phenomena belong partly to the precursors of acute diseases, whilst they are partly signs of a chronic course and of great obstinacy. The greatest intensity of the phenomena is not always at the seat of disease, those which are sympathetic being often much more violent than those proceeding from the diseased organ itself. Great severity in the phenomena in some subordinate organ warrants us more in inferring disease of the central organ, than if these phenomena had presented themselves in a slighter degree. The duration of the phenomena is of considerable importance with respect to diagnosis and prognosis. The more persevering the phenomena are, the sooner may we admit the seat of them to be in that organ which manifests them. Long duration of a phenomenon indicates great obstinacy, and warrants us in predicting continued organic changes. Alternation of the phenomena in one and the same organ indicates a rapid course. If, on the contrary, passing phenomena present themselves now in this, now in that organ, we either have to do with a morbid process which is wandering in its nature, or some central organ is the seat of the affection. If some phenomena have obstinately continued for a long time in the same form, and at length some change occurs in them, one may expect a termination of the disease. If the change is more favorable than the previous state, a hope of convalescence is afforded. The longer time favorable signs continue, the better is the prognosis. Among the forms which a functional disturbance may admit, that of exaltation is the most favorable for prognosis. Phenomena depending on exaltation of the functions indicate at the same time the simplest form of disease, a rapid course, and the participation of a limited number of organs. Diminution or total suspension of a function indicates a long duration of the disease, and depends on a serious disturbance of the organ in question, or on a modification of the nervous influence proceeding from the nervous centres, or on a change in the blood. The perversion of a function, where this no longer is performed according to physiological laws, is always a sign that the nervous system has lost its normal influence over the affected organ. The relation of time, in which the newly-occurring signs stand to the signs already present, is of the greatest moment in judging of the former. Violent morbid phenomena are not dangerous about the time of the crisis. If the critical precursors come before this period, and do not bring relief, they are of bad import. If the intensity of the morbid signs diminish according to the critical movements, the prognosis is favorable; if, in acute disease, after this diminution, new symptoms differing from the first present themselves, metastasis is to be dreaded. Diminution of the phenomena in number and intensity is a favorable sign at every period of the disease. If at the time of the crisis several peripheric organs, or organs opening externally, give signs of increased activity, it is more favorable than if this happen only in one organ. A concordance of the phenomena with respect to form is of great importance with regard to prognosis. Favorable phenomena yield a good prognosis only when they harmonize. A single bad sign in the midst of a series of favorable signs makes the prognosis unfavorable. Perversion of a function with signs of exaltation of the same is bad, but still more so, if to the signs of depression those of perversion be joined. If, on the contrary, during the existence of signs of diminished activity, some increase of the same is observed in all the organs attacked, we may hope for a favorable issue. But if this increase does not follow uniformly in all the affected organs, but, whilst the activity of one organ is increased, that of another diminishes, we are not yet to expect the termination of the disease. If the activity of peripheric organs, especially secreting organs, increases, whilst that of the secreting organs be depressed below the natural standard, the disease is to be considered dangerous. If the relation be reversed, the disease is still at the commencement, and in the stage of crudity. In case of a favorable issue the activity of the peripheric organs should increase, and that of the central organs should gradually approximate to the normal state. A contradiction in the signs of the central organs is bad, particularly between the signs afforded by the vascular and nervous systems. Want of harmony in the signs of several parts of the body is of unfavorable import. Having now followed our author through the first part of his work, in which he treats of *signs in general*, we come to the *special part*, which he divides into nine chapters; the *first* containing signs derived *from the nervous system*, as from the sensitive and perceptive faculties, and from the higher intellectual functions—the *second* treats of the signs derived *from the senses*. The *third* considers the signs derived from voluntary motion and its organs. The *fourth* treats of signs derived from the *vascular system*. The *fifth*, those derived from the *respiratory organs*. The *sixth*, those from the *skin*. The *seventh*, those derived from the *urinary organs*. The *eighth*, those from the *digestive system*; and the *ninth*, those from the *generative organs*. We shall now present our readers with some extracts from his last chapter, namely, that which treats of the signs derived from the generative organs.

### Signs from the Parts of Generation.

*Signs from the Male Parts of Generation.*—The retention of the male genital parts in the development of the body is a sign of a general state of disease interfering with and impeding nutrition, more especially of scrofula, of diseases of the stomach, lungs and brain. Extraordinary size of the penis is a sign of sexual excesses, in boys it is a sign of onanism, of calculus in the bladder, and of chronic diseases of the portal system. Unusual smallness of the same organ, and long-continued dependence of the scrotum, indicates partly local debility of the genitals after violent and repeated excesses in sexual enjoyment, and after onanism, whilst it sometimes indicates general debility of body. Those cases of smallness of the penis must be distinguished from those instances where a portion of its skin is involved in hydrocele or scrotal hernia. In violent pains in the abdomen the penis appears to be extremely small. Clear, transparent vesicles grouped on the prepuce and glans, followed by desquamation in thin, small, flat scales, and which sometimes occasion excoriations, which also form groups, are a sign of herpes præputialis. Pustules which subsequently change into elevated, thick crusts, ulcers on the penis and scrotum, and excrescences on the genital parts, are of syphilitic origin. Parts somewhat elevated which secrete small thin scales, without having been preceded by vesicles, are a sign of psoriasis. They appear on the scrotum and penis, generally however at the same time on other parts of the skin. Very small transparent vesicles which at a subsequent period run into an inflamed smarting surface, constantly secreting scales or serum, indicate eczema. If the fluid of the vesicles at first clear, then becomes turbid, the disease is eczema impetiginoides. Lastly the scrotum is the favourite seat of chimney-sweepers' cancer.

The want of testicles indicates cryptorchy and impotence. Their being drawn towards the inguinal ring occurs in violent pains in the abdomen, more especially in inflammation of the bladder, nephritis calculosa, stone in the bladder, in neuralgia ileo-scrotalis, and in cramps. Swelling of the scrotum indicates rupture, when it is drawn into the open inguinal ring, is replaceable, and a rumbling noise is heard on attempting to replace it. We shall be warranted in concluding that the case is hydrocele, if the swelling has commenced at the bottom of the sac, if it be tense and transparent, if there be fluctuation, if it occasion no pains, at least in its advanced stage, and is connected with no signs of indigestion.

If the tumor consist of soft compressible cords and diminishes in the horizontal position, it is varicocele. If a swelling of the epididymis is composed of several small vermiform cords, painful on pressure, and if it has commenced with a feeling of pressure and tension, it is spermatocele. A hard, uneven, knobby tumor with lancinating pains, characterizes scirrhus of the testicle. A uniform, painless, large tumor, simulating fluctuation and running abruptly into the abdomen, indicates the existence of medullary sarcoma (Markschwamm). A heavy, uniform, painless tumor of the form of the testicle and without being continued to the spermatic cord and into the abdomen, indicates sarcoma of the testicle. Inflammation of the testicle, and the consequent induration, thickening and serous infiltration of the cellular tissue of the scrotum, effusion of pus, or of blood into the same, a varicose state of the scrotum likewise occasion tumors, which are distinguished from the above as well by the history as by the accompanying phenomena and the feel. The testicle sometimes swells in consequence of metastasis, as for example, in parotitis, typhus, &c.

Want of erection occurs in impotence, in general debility, in diabetes, and in some chronic diseases of the brain. Moderate erections indicate in fever patients the commencement of convalescence. Too frequent erections when the external occasions are inconsiderable, and even without any such, are a sign of irritation of the sexual organs through onanism, or sexual abuses, or through inflammation and ulceration on the penis, or irritation occasioned by stone in the bladder, acrid urine, and ascarides of the rectum. Continued erection (priapism) is sometimes the consequence of deficient seminal secretion; it is however most commonly occasioned by catarrhs, inflammations and hæmorrhages from the bladder, stones in the bladder and kidneys, by gonorrhœa; it has also been observed in diseases of the cerebellum, in epilepsy, hypochondriasis, erotic melancholy, and in many cases of acute diseases attended with great debility.

It becomes painful in cases where inflammation of the penis, or bladder, or where gonorrhœa is the cause, more especially when the concavity of the penis looks downwards (chordée).

The secretion and excretion of semen presents several deviations from the normal state. Deficiency in the seminal secretion is a sign of atrophy of the testicle, of disease of both testicles, and of impotence. Excessive seminal secretion is a sign of plethora and of irritation of the genitals. Involuntary discharge of semen by day, without erection, during riding or whilst evacuating the rectum or bladder, is a sign of debility of the genitals and of dilatation of the ejaculatory ducts. In tabes dorsalis it gives an unfavourable prognosis. Discharge of semen at the commencement of an erection is a proof of debility of the genital organs, as it is produced by sexual excesses.

Defective ejaculation of the semen is a sign of debility of the genitals, or of mechanical obstructions, as strictures of the urethra, narrowness in the orifice of this canal, hypospadia, &c.

Want of sexual desire is natural at the commencement of all acute diseases, it is further produced by degenerescences and atrophy of both testicles, by great debility and cerebral disease.

*Signs from the Female Parts of Generation.*—The female genital parts are much more intimately connected with the entire system than the corresponding parts in the male. Whilst the latter stand in close relation only to the nervous system, an intimate reciprocal action is found to exist in the female genitals with the sanguineous system and the system of nutrition. The signs which may be obtained from them, may be referred to the condition of the organs, the sexual inclination, and the secretions from the female genitals.

A retardation in the development of the female genitals after puberty is either a sign of deficiency or atrophy of the ovaries, or it is occasioned by chronic diseases, particularly scrofula and rachitis.

A proportionally too wide vagina is a sign of onanism, of the fact of coition having taken place, of the births of children, of prolapsus of the vagina and uterus. Ulcerations and warty excrescences are a sign of syphilis: eczema and psoriasis also come on the female genitals. Swelling of the labia may be occasioned by inflammation (sometimes the result of metastasis in erysipelas and parotitis) by œdema, extravasated blood, &c.

Uneven hardness of the cervix uteri indicates scirrhus ; ulcers indicate syphilis, scrophula, and cancer of the same. Pain on touching the part, elevated temperature and great softness, are signs of metritis. When the uterus is in a state of inflammation, it presents an increase of weight, but in a higher degree however in degenerescences, morbid growth, and in pregnancy.

If the os uteri is round, there is a morbid tumor on the cavity of the uterus, unless pregnancy or menstruation be the cause. If a body presses forth from it, this, unless parturition is commencing, must be the retroverted uterus, or polypus. Fungous growths at the os uteri are a sign of cancer. The changes in the direction of the cervix uteri denote dislocations of the uterus. They are occasioned by relaxations of the ligaments of the uterus, degenerescences at the fundus of the organ, and pressure of some neighbouring organ in a state of degenerescence.

Menstruation affords at its commencement, by the accompanying phenomena and the quantity and quality of the secretion, very important signs. A too late appearance of this secretion may be occasioned as well by chronic diseases as by a laborious mode of life. Too early occurrence of menstruation is a proof of an early perfection of growth. If its appearance is too long retarded, either an organic malformation, such as absence of the uterus, imperfect vagina or of the os uteri, or great debility must be the cause.

If menstruation be accompanied with great pain, spasms, nausea and vomiting, there is present either a local morbid state of the sexual organs, as degenerescence of the uterus, considerable narrowing of the vagina, chronic inflammation and degenerescence of the ovaries, or hyperemia of the intestinal canal, the liver and spleen are enlarged, or finally general debility may be the cause of the painful menstruation. Morbid retention of the menses occurs in most diseases affecting the entire system, particularly in fevers, then in inflammations, in diseases also accompanied with spasm, in tubercles of the lungs or of the intestinal canal, also in metritis, in degenerescences and morbid growths affecting the internal genitals. Retention of the menses may be followed by congestions to other parts, more especially to the intestinal canal, to the lungs and to the brain, which may ultimately give rise to inflammation, hemorrhage, morbid secretions, and disturbances of the nervous system. The consecutive states occur in a much more acute form in suppression of the menstruation.

Thick, dark menstrual blood is a sign of disturbance and plethora of the intestinal canal, engorgement of the spleen, disease of the portal system, unless it acquires this property by retention of the discharge. It is pale and thin in chlorosis, anæmia, phthisis, and dropsy. The secretion is scanty in the lower grades of those diseases which occasion amenorrhœa, and also in hyperemia of the intestinal canal, in what are called infarctions and in hemorrhoids. Too profuse and too long-continued menstruation is a sign of plethora, of increased vitality of the sexual organs at the expense of the other organs, or of great debility of the same ; it is critical in metritis, it is also observed in intestinal inflammations, in chronic inflammation of the ovaries, and in ulceration of the uterus. It may be followed by abortion in pregnancy, also by hysteria, degenerescences of the generative parts, more particularly of the ovaries and uterus.

We shall now close our analysis of this work, not however without expressing how much we have been gratified by its perusal. The merits of the book, and the great desideratum of such a work in our own language, render it not improbable that we shall see this manual ere long in an English dress.

